# The Impact of Personalized Human Support on Engagement With Behavioral Intervention Technologies for Employee Mental Health: An Exploratory Retrospective Study

**DOI:** 10.3389/fdgth.2022.846375

**Published:** 2022-04-27

**Authors:** Jehanita Jesuthasan, Megan Low, Tiffanie Ong

**Affiliations:** Naluri Hidup Sdn Bhd, Kuala Lumpur, Malaysia

**Keywords:** occupational health, mHealth, mental health, Employee Assistance Program (EAP), digital mental health intervention, Supportive Accountability

## Abstract

Digital healthcare has grown in popularity in recent years as a scalable solution to address increasing rates of mental illness among employees, but its clinical potential is limited by low engagement and adherence, particularly in open access interventions. Personalized guidance, involving structuring an intervention and tailoring it to the user to increase accountability and social support, is one way to increase engagement with digital health programs. This exploratory retrospective study therefore sought to examine the impact of guidance in the form of personalized prompts from a lay-person (i.e., non-health professional) on user's (*N* = 88) engagement with a 16-week Behavioral Intervention Technology targeting employee mental health and delivered through a mobile application. Chi-squared tests and Mann-Whitney tests were used to examine differences in retention and engagement between individuals who received personalized prompts throughout their 4-month program and individuals for whom personalized prompts were introduced in the seventh week of their program. There were no significant differences between the groups in the number of weeks they remained active in the app (personalized messages group *Mdn* = 3.5, *IQR* = 3; control group *Mdn* = 2.5, *IQR* = 4.5; *p* = 0.472). In the first 3 weeks of the intervention program, the proportion of individuals who explored the educational modules feature and the messaging with health coaches feature was also not significantly associated with group (*ps* = 1.000). The number of modules completed and number of messages sent to health coaches in the first 3 weeks did not differ significantly between the two groups (*ps* ≥ 0.311). These results suggest that guidance from a non-health professional is limited in its ability to increase engagement with an open access Behavioral Intervention Technology for employees. Moreover, the findings suggest that the formation of a relationship between the individual and the agent providing the guidance may be necessary in order for personalized guidance to increase engagement.

## Introduction

About 20% of the workforce suffers from a mild or serious mental or psychiatric disorder (1), and the prevalence of mental illness and work-related stress has been rising in recent years (2). This has brought about high individual, societal, and economic costs, including increased sickness absence (3), early retirement (4, 5), and reduced productivity (6). Digital mental health interventions for employees, which target large-scale, long-term wellness and health management, have the potential to minimize barriers to engagement with in-person psychological treatments, including shortages of mental health professionals, lack of flexibility, and stigma associated with mental illness (7). Indeed, evidence indicates that these approaches can be effective in reducing mental stress, depression, and psychological distress levels among employees (8, 9).

Behavioral Intervention Technologies [BITs; (10)] are a subset of digital mental health interventions and are designed to improve mental health through behavior change. Previous research has demonstrated that digital interventions rooted in behavior change theory are particularly effective (11), including in occupational settings (12). Crucially, success in these interventions, defined as engagement in health behaviors, is contingent on engagement with features and components of the digital intervention itself (13, 14). However, despite the recent growth in digital healthcare solutions and their clinical potential, completion rates for digital health programs tend to be low, and limited user engagement and adherence is one of the biggest challenges in maximizing their impact (15, 16). Indeed, intervention completion for web-based employee psychological interventions can be as low as 3% (8) and a cross-study evaluation reported a median retention of 5.5 days of app usage in digital health studies (17). To increase the effectiveness of self-directed mental health support delivered digitally, it is therefore essential to identify solutions that sustain engagement.

Guidance, involving structuring of the intervention or reminders to use it, has been identified as one of the program-related factors that promotes engagement with such BITs involving content for users to engage with independently (18–20). Interventions that include guidance are associated with better outcomes, including greater symptom improvement and intervention completion rates, than unguided interventions (10, 21, 22). Recipients of digital health interventions report that text message prompts increase their perceived support, enhance their motivation for healthy behavior change, and encourage their engagement in health behaviors (23). While such guidance can be machine-driven, for example through automated engagement monitoring and push notifications, human support can increase adherence to a greater degree (24, 25). According to Mohr et al.'s (26) Supportive Accountability model, this is because the human component provides accountability and social support. Indeed, accountability–the expectation that one may be called upon to justify their actions or inactions (27)–encourages goal-directed behaviors (28), and requires the social presence of another human. Moreover, in self-directed digital interventions where most of the content of the treatment is not delivered by a therapist, the social support provided by human guidance may help compensate for the absence of a therapeutic alliance and maintain engagement.

In addition, tailoring of interventions has been shown to improve outcomes in behavior change interventions (29). Tailoring refers to the personalization of an intervention's characteristics, such as content or timing, to increase engagement by increasing heuristic and self-referential processing, and reducing effortful processing, to increase the end user's receptiveness (30). Accordingly, personalized activity recommendations combined with leeway for autonomous choice in activities leads to higher engagement with BITs by providing clear and personally relevant guidance for how to use the intervention while also allowing for exploration and spontaneous use of the intervention (31). Engagement is a crucial target in open access workplace BITs, as observational studies of digital health interventions and open access interventions have higher attrition rates than randomized controlled trials (32, 33).

The current study examined the impact of human guidance and support from a lay person (i.e., non-health professional), in the form of regular, personalized prompts to use app features sent via in-app text messages, on retention and engagement in an Employee Assistance Program (EAP) involving a BIT delivered through an app (Naluri). Naluri is a digital therapeutics solution that offers personalized 16-week health programs based on the principles of behavior change. The employee BIT program aims to improve psychological wellbeing through psychoeducation and skill training. Specifically, the program includes educational modules, a health journal for self-monitoring, and a habit formation and tracking feature, as well as one-on-one coaching from health professionals (including psychologists, dietitians, fitness coaches, and medical advisors) via text-based messages and video calls.

This study focused on the effectiveness of tailored prompts from a non-health professional (the Naluri Assistant) in increasing retention and engagement with the BIT content, specifically the educational modules and text-based exchanges with health professionals. In this study, engagement was defined as exploration of the app's features and number of activities in each of these features.

## Methods

### Design and Participants

This was a retrospective study using data from the digital therapeutics company Naluri (Naluri Hidup Sdn. Bhd., Kuala Lumpur, Malaysia) to examine the effects of support from a lay person on app engagement over a 16-week digital health program. Participants included in the study registered for an account on the app voluntarily through sponsorship from their employer (Company A).

Participants in the personalized message group were Naluri app users in Malaysia who took part in Naluri's 16-week EAP offering and received targeted weekly messages from the Naluri Assistant throughout their time using the app (*N* = 30).

Participants in the control group were employees of the same company who also joined through their employer but did not receive the personalized messages from the Naluri Assistant until the seventh week of their program (*N* = 75). Participants in this group only began receiving the targeted messages later in their program as the personalized messaging curriculum was not yet introduced at their time of joining. The unequal group sizes are due to the observational nature of the study and a larger number of employees joining the Naluri app before the personalized prompts curriculum was developed and introduced.

Eligibility criteria for the Naluri EAP was: moderate or above scores on the Depression, Anxiety and Stress Scale (DASS-21) (34) in a company-wide mental health screening or used at least one app feature within the first 2 weeks after registering with Naluri.

Participants who did not select a health goal upon joining the application or who selected “Just Exploring” as their reason for using the app were excluded from the analyses. The health goal options included “Managing Stress and Depression,” and “Improving General Health,” for example. The final sample sizes were therefore *N* = 28 for the personalized messages group and *N* = 60 for the control group. Participant characteristics are shown in Table 1.

**Table 1:** Participant characteristics.

		**Personalized messages (*N* = 28)**	**Control (*N = 60)***
Age (mean ± SD)		39.68 ± 8.43	39.41 ± 8.98
Gender (%)	Male	35.71	50.00
	Female	32.14	26.67
	Unknown	32.14	23.33
Psychological symptoms (%)	Low	32.14	58.33
	Mild to moderate	25.00	18.33
	Severe to extremely severe	17.86	13.33
	Unknown	25.00	10.00

*Low severity of psychological symptoms is defined as a depression score < 10, anxiety score < 8, and stress score < 15. Severe to extremely severe symptom severity is defined as a depression score ≥ 21, anxiety score ≥ 15, and/or stress score ≥ 26. Mild to moderate symptom severity refers to scores lying between these values for depression, anxiety, and stress, respectively*.

This study received ethical approval from the Malaysian Medical Research and Ethics Committee (Research ID #60291).

### Materials

#### Measures

The DASS-21 (34) was used to assess participant's mental health before beginning the Naluri digital EAP. The DASS-21 is a 21-item self-report questionnaire that includes three subscales designed to measure the severity of depression, anxiety, and stress symptoms, respectively. The depression subscale measures symptoms corresponding to hopelessness, anhedonia, dysphoria, and low energy. The anxiety subscale includes items related to autonomic arousal as well as situational anxiety and subjective experience of anxious affect. The stress subscale relates to chronic non-specific arousal, irritability, nervousness, and tension. Each of the seven items on the three subscales has four response options ranging from 0 (*did not apply to me at all*) to 3 (*applied to me very much, or most of the time*). The questionnaire is scored by summing the items for each subscale and multiplying the results by a factor of two, yielding a score between 0 and 42 for each subscale. These scores can be classified into five categories using the cut-offs proposed by Lovibond and Lovibond (34): normal, mild, moderate, severe, and extremely severe. Symptoms of moderate or above depression, anxiety, and stress, used as a threshold for eligibility for the Naluri program, are defined as scores ≥14, ≥10, and ≥19, respectively.

The DASS-21 has been shown to have strong reliability and validity for measuring depression, anxiety, and stress among a working population (35). Moreover, the measure has moderate to high correlation with other common self-report measures of mental health, specifically the 9-item Patient Health Questionnaire [PHQ-8; (36)] and the 7-item Generalized Anxiety Disorder scale [GAD-7; (37)] in virtual behavior healthcare settings (38). However, the PHQ-8 and PHQ-9 (39) and the GAD-7 tend to classify a higher proportion of individuals as having above threshold symptoms of depression and anxiety symptoms than the DASS-Depression and DASS-Anxiety scales, respectively (38, 40).

#### Naluri Digital Employee Assistance Program

This study used the Naluri app, a BIT delivered to employees in the form of 16-week programs. Naluri's mental health program is built on principles of behavior change and the transtheoretical model of change (41) and aims to improve psychological wellbeing through psychoeducation and skills training. Specifically, the BIT supports individuals in attaining specific outcomes (such as improved relationships and social skills, or development of healthy habits) to in turn improve their mental health. Naluri's program is delivered through a combination of independent activities/features (educational modules, a health journal, and a habit tracker) and a team of professional health coaches who are in contact with users primarily through in-app text message channels, as well as video calls.

The educational modules cover a range of health and wellbeing-related topics, including mental wellbeing (e.g., healthy coping, relaxation techniques and cognitive restructuring), healthy eating, exercise and movement, and implementing healthy habits. The health journal includes a thought journal in which users can log both positive and negative thoughts and moods, and optionally share these with their health coaches. The health journal also includes a food journal to which users can upload pictures of their meals and receive feedback on them from qualified dietitians. The habit tracker feature allows users to set habits and receive reminders to complete these at the set interval.

The multidisciplinary coaching team is led by a qualified clinical psychologist and includes dietitians, physiotherapists, fitness coaches, pharmacists, and medical advisors. Each coach holds the necessary qualifications to practice in their area (i.e., a clinical psychology Doctorate/Master's for psychologists, a dietetics degree for dietitians, a physiotherapy degree or diploma for physiotherapists, a personal trainer certification for fitness coaches, a pharmacy degree for pharmacists, and medical degrees for medical advisors). All coaches also completed additional training on digital coaching with Naluri, which focused on how to use health psychology techniques in a digital context to help users improve their mental and physical health through behavior change.

Alongside the self-directed features, Naluri coaches provide one-on-one support to participants in the following areas: identifying and understanding their emotional distress; identifying the psychological, social, and individual factors contributing to this distress; psychoeducation and skills training in areas including self-care, problem-solving, emotion regulation, and other coping skills; and building resilience and maintaining healthy habits. Throughout the program, the health coaches apply motivational interviewing techniques and cognitive behavioral therapy to support participants in their goal setting and habit formation. The coaches reach out to participants at the beginning of the 16-week program to introduce themselves and explain their role as a coach. Subsequently, the coaches engage in text-based conversations with participants as and when the latter initiate exchanges.

The Naluri app also includes a Naluri Assistant, who is a non-health professional that users can reach out to for support in using and understanding the app and its features. This Naluri Assistant does not hold professional health qualifications but is trained in psychological first aid and in moderating peer support groups. The Naluri Assistant periodically sends app users messages regarding new features or changes within the app. The Naluri Assistant, as well as each coach, can be reached through their dedicated text-based messaging channel within the app. While the focus of the coaching is to deliver parts of the intervention and provide personalized therapeutic content, the role of the Naluri Assistant is to provide practical support with accessing and using the different elements of the digital intervention. These two distinct sources of human support within the Naluri digital health program enables participants to receive personalized professional coaching, as well as a source of support available to help address usability and engagement failures (42) that is more scalable as it does not require a trained health professional.

#### Personalized Messaging Curriculum

The personalized messaging was a pilot developed to facilitate familiarization with the app and encourage engagement in the context of remote on-boarding to the Naluri program due to social distancing measures during the COVID-19 pandemic. Prior to this, in-person onboarding allowed first-hand demonstration of the app to kickstart engagement with the program. The personalized prompting involved in-app text-based messages delivered by the Naluri Assistant to prompt participants to complete activities within the app. The messages followed a 16-week curriculum and the prompted activities corresponded to topics identified by Naluri psychologists as commonly discussed issues on the platform. These topics included identifying one's intrinsic motivations, setting health and behavior goals, emotion regulation, and building resilience. The sequence of topics followed the Naluri coaching model through the phases goal-setting, implementation of action, and finally, maintaining learned practices.

At least one message was sent by the Naluri Assistant each week, introducing the week's topic and prompting the completion of a relevant app feature, such as messaging their psychologist or completing a module. Every introduction message was sent with an infographic of the topic. Topic introduction messages from the Naluri Assistant were standardized across participants, while follow-up messages sent later in the week were personalized to the participant's in-app activity. For personalization, variations of messages were scripted to take into account every possible pairing of incomplete and/or completed prompts, and to mirror the participant's preferred messaging style. Throughout the course of the personalized messaging, incomplete activities were prompted while completed activities were congratulated and acknowledged. Thus, in line with the Supportive Accountability model (26), the Naluri Assistant aimed to develop a bond with the participants through regular messaging, and to set up accountability structures, by prompting specific activities and monitoring their engagement with the app. However, in contrast to this model, the Naluri Assistant also delivered some intervention content, in the form of an infographic providing an overview of the subject area that was the focus of the week's suggested activities.

### Procedure

Participants were referred to the Naluri app through their employer (Company A). During a virtual company-wide event organized by Naluri, employees from Company A completed an online mental health screening that consisted of the DASS-21. Upon completion of the questionnaire, employees received their results and were prompted to create a Naluri account and download the application via a link that was provided. Employees were therefore able to register with Naluri immediately after the health screening and continued to be able register for 3 months after the health screening event. The personalized messaging program was launched approximately 1.5 months after the health screening. Thus, participants in the control group registered within a week after the health screening, while participants in the personalized messages group registered 1.5 to 3 months after the health screening. Upon beginning the program, the mental health symptoms severity scores were therefore more recent among participants in the control group than among participants in the personalized messages group.

After registering with the application, participants created a profile in which they indicated their age and gender and were asked to indicate their reason for using the application (i.e., their health goal). Participants were provided with a range of options from which to select their health goal, including “Managing Stress and Depression,” “Improving General Health,” or “Just Exploring.” Following this, participants were able to use any of the apps features as they preferred.

### Data Analysis

#### Objectives

The objective was to compare app usage between users who received personalized prompts over their 16-week health program and users who did not receive these prompts. App usage was compared in three areas: length of retention, rate of feature exploration (i.e., engaging with the feature at least once), and total number of messages sent to coaches and modules completed. Messages sent to the Naluri Assistant were used as a proxy for receipt and acknowledgment of the prompts and were also compared between groups. Length of retention was defined as the number of weeks between the participant's joining date and their last in-app activity.

#### Statistical Analyses

Data for retention length and activity counts are presented in median (Mdn) and interquartile range (IQR). The categorical variable exploration rate for individual features is reported in percentages. For comparing length of retention over the 16-week health program, Mann-Whitney tests were used, as the data was not normally distributed and because non-parametric tests are more robust to unequal group sizes. Similarly, Mann-Whitney tests were used to compare total activity count, as well as number of modules completed, and number of messages sent to coaches and to the Naluri Assistant between the two groups. The associations between group and exploring the modules as well as the chat with coaches feature were examined using Chi-squared tests. To account for low retention rates in digital health interventions (17, 43), engagement metrics were only analyzed for the period of the program during which at least 50% of the participants were still active in the app (i.e., for the sample's median length of retention). The analyses were conducted using statistical software *RStudio* version 1.3.1056.

## Results

### Retention Length Between Groups

In the personalized messages group, 15.00% of participants were still active at the halfway point of the program (in week 8), and 5.00% were still active in week 16. In the control group, 10.71% were still active by week 8, while 3.57% were still active in the final week. Across the sample, the median retention length was 3 weeks. There was no significant difference in retention length between the personalized message group (*Mdn* = 3.5, *IQR* = 3) and the control group (*Mdn* = 2.5, *IQR* = 4.5; *U* = 760.5, *p* = 0.472).

### Percentage of Participants Engaging With Features

As the median retention length was 3 weeks, exploration of features during the first 3 weeks of the Naluri program was examined. The percentages of participants in each group who used the modules feature and coaches chat feature at least once in this period are shown in Table 2. There was no significant association between group and attempting modules at least once [*X*^2^(1) < 0.01, *p* = 1.00] or between group and messaging coaches [*X*^2^(1) < 0.01, *p* = 1.00].

**Table 2:** Percentage of participants who engaged in each feature at least once in weeks 1 to 3.

	**Modules^**a**^ (%)**	**Messages to coaches (%)**
Personalized messages	82.14	28.57
(*N* = 28)
Control (*N* = 60)	81.67	28.33

a *The Naluri app included 97 modules available to the participants*.

### Activity Count per Participant

In the first 3 weeks of the program, the median number of in-app activities completed by participants (Table 3) did not differ significantly between groups (*U* = 941.0, *p* = 0.365). There was also no significant between-group difference in the number of modules completed (*U* = 951.5, *p* = 0.311), messages sent to coaches (*U* = 844.0, *p* = 0.969), or messages sent to the Naluri Assistant (*U* = 825.0, *p* = 0.805).

**Table 3:** Median number of activities per user in each feature in weeks 1 to 3.

	**Total activity *Mdn (IQR)***	**Modules^**a**^** ***Mdn (IQR)***	**Messages to coaches *Mdn (IQR)***	**Messages to Naluri Assistant** ***Mdn (IQR)***
Personalized messages	3.00 (5.00)	1.00 (1.25)	0.00 (1.25)	0.00 (0.00)
(*N* = 28)
Control (*N* = 60)	4.00 (9.50)	2.00 (3.00)	0.00 (1.00)	0.00 (0.00)

a *The Naluri app included 97 modules available to the participants*.

## Discussion

The current study set out to examine the effectiveness of personalized prompts providing guidance on increasing employees' engagement with an employer-sponsored Behavioral Intervention Technology (BIT). There was no significant difference in length of retention or number of in-app activities in the first 3 weeks of the program between those who received personalized messages in that period and those who did not. In addition, the proportion of participants completing at least one interactive educational module or reaching out to health coaches over the first 3 weeks of the program also did not differ significantly between the control and personalized prompts groups. These findings suggest that the personalized guidance in the form of prompts used in this study is limited in its ability to overcome barriers to engagement in open-access preventative mental health interventions for employees.

The findings that participants who received guidance did not engage with the BIT more than participants who did not receive guiding prompts are in contrast with findings from previous studies on BITs (18). The low rates of reaching out to coaches may be explained by a high barrier to reaching out for help for psychological problems in our sample. Indeed, studies have identified a preference for self-reliance and a desire to handle the problem on one's own, as well as stigma of mental illness–which is prevalent in Malaysia, where our sample was from–as common reasons for not seeking treatment for poor mental health (44–46). Mojtabai et al. (44) also reported that low perceived need for treatment was a common barrier to seeking treatment, particularly among individuals with mild mental disorders. This barrier is especially relevant in the current study, in which 25% of the participants receiving personalized messages were identified as having mild to moderate symptoms of psychological distress, as measured by the DASS-21 (34). This group are likely to have been using the app with the goal of improving their positive psychological wellbeing, and the absence of more serious psychological distress may have made them more reluctant to reach out to a health professional, in spite of guidance to do so.

The lack of differences between groups in this study may also be due to the nature of the relationship between the Naluri Assistant and the participant, needed in order for accountability and social support to be felt by the user. Indeed, engagement with the Naluri Assistant did not differ significantly between participants in the two groups. This may be because the guidance messages directed participants to other app features, rather than prompting a reply to the message, thus encouraging the perception of the Naluri Assistant as a guide rather than a conversational agent providing social support and with whom users should interact. As a result, the prompt messages may be regarded in the same way as automatic notifications, and lose the benefit of guidance from a human compared to automated reminders outlined by the Supportive Accountability model (26). Indeed, when the source of a prompt is perceived to be automated rather than human, individuals are less likely to attend to it (30). Prompts from a human also tend to be more effective when they are accompanied by a two-way interaction between the agent delivering the prompts and the user, as this allows for a stronger alliance to be formed, which in turn promotes engagement (47). A greater emphasis on eliciting conversation with the participant in the messages may therefore have had a greater impact on increasing engagement with the prompted activities.

The absence of a significant difference in the length of retention between the control group and the personalized messages group suggests that the personalized prompts used in this curriculum were also insufficient to sustain continued engagement. This may be due to habituation or ignoring prompts, which are likely to occur in prompt-based interventions unfolding over the long-term (30). Although there is no research directly examining habituation in digital patient-centered health interventions, alert fatigue has been found to occur in studies examining responsiveness to alerts among health providers (48–50). The lack of effectiveness for the personalized prompts could suggest that the guidance curriculum requires additional refinement in order to be more effective and maintain interest over time. While the prompts were tailored to the individual's previous in-app activities and their messaging style, they were not tailored to the individual's health goals or health status and instead prompted in-app activities that were general and applicable to all types of health goals. This may have resulted in a low perceived fit among participants, and the addition of further information relevant to their current situation may improve the prompt's effectiveness (18).

### Limitations

This study has several limitations which should be taken into account when considering its findings. First, the sample size for the personalized messages group was small and the groups were of unequal sizes because this was a retrospective study in which participant's groups were determined by the timing of their joining. While the non-parametric statistical tests used are robust to unequal group sizes, the results should nonetheless be interpreted with caution. Second, participants enrolled in the Naluri EAP were those who reported experiencing moderate or above scores of depression, anxiety, or stress on the DASS-21 in the health screening or who started using the app within the first 2 weeks after registering with Naluri. However, individuals with elevated levels of depression, anxiety, or stress may have different app usage patterns than individuals who do not experience such symptoms and are merely using the app in a casual or exploratory manner. To mitigate against within group differences, though, only individuals who provided a health goal as a reason for joining the app (rather than just exploring the app) were included in the study sample. Consequently, all of the participants indicated that they were using the app the achieve mental health symptom reduction or health improvement, reducing the within-group heterogeneity. Third, receiving the prompts was contingent on having notifications turned on for the app, and it is possible that this was not the case for all participants, who may therefore not have seen the prompts. While the inclusion of all participants regardless of their notification settings provides insights into the real-world effectiveness of personalized messaging, it does not allow for an examination of the efficacy of such prompting.

### Conclusions

This preliminary and exploratory study provides insights into the effectiveness of human support in increasing engagement with digital health interventions in real-world conditions. Overall, the lack of differences between individuals who received personalized support and those who did not suggests that, to increase retention or engagement in Behavioral Intervention Technologies delivered in open-access Employee Assistance Programs, human guidance and support programs must incorporate certain elements that were not included here. For example, the formation of a relationship between the individual and the agent providing the guidance seems to be a critical element in the effectiveness of such support. Additional work is needed to identify the elements involved and to in turn refine personalized guidance curriculums.

## Data Availability Statement

The raw data supporting the conclusions of this article will be made available by the authors, without undue reservation.

## Ethics Statement

The studies involving human participants were reviewed and approved by Medical Research and Ethics Committee (Malaysia). Written informed consent for participation was not required for this study in accordance with the national legislation and the institutional requirements.

## Author Contributions

ML and TO proposed and designed the study. JJ made contributions to the design of the work and performed the statistical analysis. ML helped acquire the data. JJ and ML interpreted the analyses. JJ wrote the manuscript with contributions from ML. All authors approved the final version of the manuscript.

## Funding

This study was funded by Naluri Hidup Sdn Bhd.

## Conflict of Interest

JJ, ML, and TO were employed by Naluri Hidup Sdn Bhd, which created the mobile application used in the study.

## Publisher's Note

All claims expressed in this article are solely those of the authors and do not necessarily represent those of their affiliated organizations, or those of the publisher, the editors and the reviewers. Any product that may be evaluated in this article, or claim that may be made by its manufacturer, is not guaranteed or endorsed by the publisher.
